# Improved Stability and Controllability in ZrN-Based Resistive Memory Device by Inserting TiO_2_ Layer

**DOI:** 10.3390/mi11100905

**Published:** 2020-09-29

**Authors:** Junhyeok Choi, Sungjun Kim

**Affiliations:** Division of Electronics and Electrical Engineering, Dongguk University, Seoul 04620, Korea; junhyeok.choi619@gmail.com

**Keywords:** memristor, neuromorphic computing, resistive switching, zirconium nitride

## Abstract

In this work, the enhanced resistive switching of ZrN-based resistive switching memory is demonstrated by embedding TiO_2_ layer between Ag top electrode and ZrN switching layer. The Ag/ZrN/n-Si device exhibits unstable resistive switching as a result of the uncontrollable Ag migration. Both unipolar and bipolar resistive switching with high RESET current were observed. Negative-SET behavior in the Ag/ZrN/n-Si device makes set-stuck, causing permanent resistive switching failure. On the other hand, the analogue switching in the Ag/TiO_2_/ZrN/n-Si device, which could be adopted for the multi-bit data storage applications, is obtained. The gradual switching in Ag/TiO_2_/ZrN/n-Si device is achieved, possibly due to the suppressed Ag diffusion caused by TiO_2_ inserting layer. The current–voltage (I–V) switching characteristics of Ag/ZrN/n-Si and Ag/TiO_2_/ZrN/n-Si devices can be well verified by pulse transient. Finally, we established that the Ag/TiO_2_/ZrN/n-Si device is suitable for neuromorphic application through a comparison study of conductance update. This paper paves the way for neuromorphic application in nitride-based memristor devices.

## 1. Introduction

Resistive switching behavior, where a dielectric layer exhibits sudden change in resistance as a result of applied electric field was first observed in oxide materials in 1962 [1]. Later, the relationship between charge and flux-linkage was theoretically revealed in 1971 [2]. For a long time, resistive switching did not receive much attention, since the researchers and engineers did not find suitable applications. However, since the 2000s, there has been increased interest by semiconductor industry and academia on resistive switching memory, due to their excellent endurance [3], retention [4], scalability [5], low voltage [6], and low current operation [7], fast switching [8], and non-volatile property in variety materials. The basic configuration of resistive memory includes two-terminal capacitor-like structure of metal–insulator–metal (MIM) [9]. There are two main types of switching (unipolar and bipolar). In the unipolar switching operation, the SET (high-resistance state (HRS) to low-resistance state (LRS)) and RESET (LRS to HRS) processes are determined by the voltage amplitude at the same polarity [10]. The SET process completes when the conducting paths are formed in the insulator and the RESET process occurs due to Joule heating as a result of high current [10]. Conversely, the SET and RESET processes of the bipolar switching occur at different voltage polarities. The movement of oxygen vacancies [11] and the diffusion of electrochemically active metals such as Ag [12] and Cu [13] due to the electric field are the driving force that causes the reversible resistive switching for the intrinsic and extrinsic switching of metal oxide, respectively.

In the near future, energy-efficient neuromorphic systems embedding resistive memory are expected to be able to replace the von Neumann architecture [14,15]. Neuromorphic computing, which emulates the neural network of the human brain, consists of a neuronal device, which acts as a processor and a synaptic device that acts as a memory connected in parallel, and can process a large amount of data with low energy consumption without big lag [14,15]. The analogue switching of the resistive memory as in the synaptic device in neuromorphic system is required to implement multiple weights [16]. Therefore, the gradual switching in SET and RESET process in resistive memory is essential in the implementation of neuromorphic device. Recently, Ag-based programmable metallization cell (PMC) has attracted interest as a neuromorphic device due to its versatile memory properties, including volatile and non-volatile switching with low power consumption [17]. The insulating layer is crucial for both intrinsic and extrinsic resistive switching memory. In the case of intrinsic switching, the insulating material is the source of conducting defects and in the extrinsic switching like PMC, the insulating material acts as the host dielectric layer. Moreover, the insulating layer like AgGeS can serve as the source of conducting filament [18]. As yet, metal oxides such as HfO*_x_* [19] and TaO*_x_* [20,21] are the leading dielectric materials owing to their excellent memory performances in regards to endurance, retention, variability, and switching speed, in addition to compatibility with complementary metal–oxide–semiconductor (CMOS). As one of the other candidates, nitride-based resistive memory, such as SiN [7,22,23,24,25], ZrN [26,27], AlN [28,29], and BN [30], has also been reported to have good memory performances.

In this work, we present Ag/TiO_2_/ZrN/Si resistive memory for non-volatile memory and neuromorphic applications. The unstable resistive switching, including negative-SET behavior of Ag/ZrN/Si device, which is investigated through DC sweep and pulse transient response. The enhanced stability and controllability of resistive switching are demonstrated by embedding 4 nm thick TiO_2_ layer to act as a buffer layer of Ag diffusion. In addition, the enhanced synaptic properties, such as potentiation and depression are verified via multiple identical pulse inputs for Ag/TiO_2_/ZrN/Si with the adoption of analogue switching for neuromorphic application.

## 2. Materials and Methods

The Ag/TiO_2_/ZrN/Si device was fabricated as follows: First, a 200 nm–thick n-type Si was deposited via low-pressure chemical vapor deposition (LPCVD) by reacting SiH_4_ and PH_3_ on SiO_2_/Si substrate. A 30 nm–thick ZrN dielectric was deposited by pulsed DC sputter, at room temperature, using a power of 0.3 kW. The flow rates of argon (Ar) and nitrogen (N_2_) were 8 sccm and 12 sccm, respectively. A deposition of 4 nm–thick TiO_2_ film was done through atomic layer deposition (ALD), using precursors of titanium tetraisopropoxide (TTIP) and H_2_O at 200 °C. A 100 nm–thick Ag top electrode was deposited via DC sputtering on the TiO_2_ layer, using a shadow mask with circular pattern of 100 μm diameter. The Ag/ZrN/Si device was prepared in the same way, except for ALD TiO_2_ deposition. The electrical properties in the DC sweep and transient modes were obtained by using a semiconductor parameter analyzer (Keithley 4200-SCS and 4225-PMU ultrafast module, Keithley Instruments, Solon, OH, USA). During the measurements, the bias voltage and pulse were applied to the Ag top electrode, while grounding the doped-poly Si bottom electrode. XPS depth analysis was conducted, using a Nexsa (ThermoFisher Scientific, Waltham, MA, USA) with a Microfocus monochromatic X-ray source (Al-Kα (1486.6 eV)), a sputter source (Ar^+^), an ion energy of 1 kV, a sputter area of 1 mm × 1 mm, a sputter rate of 0.3 nm/s, and a beam size of 100 μm.

## 3. Results and Discussion

**Figure 1:**
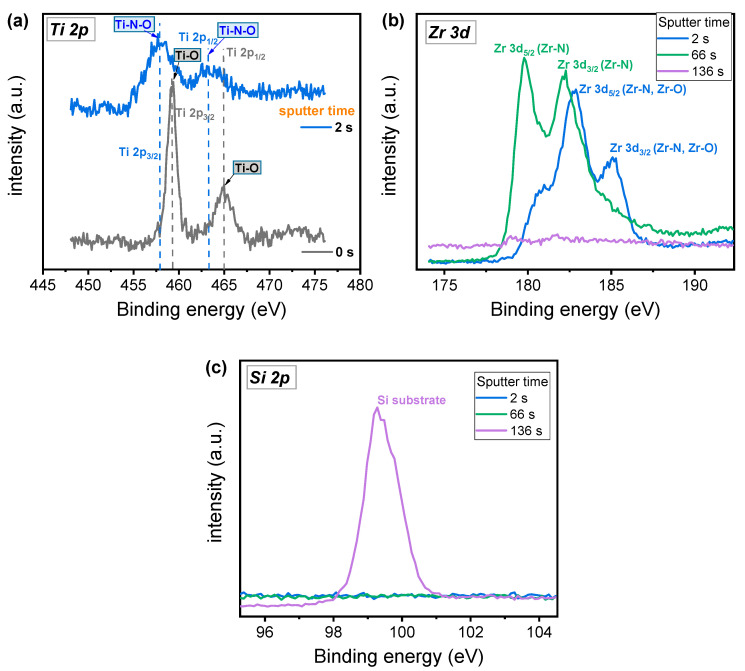
XPS spectra of (**a**) Ti 2p, (**b**) Zr 3d, and (**c**) Si 2p obtained by TiO_2_/ZrO_2_/Si depth profile.

XPS analysis was utilized to confirm the deposition of TiO_2_/ZrN/Si layers. The analysis did not include Ag, since it is difficult to control etching time. Figure 1a shows the Ti 2p spectra with different sputter time. The peak binding energies of Ti 2p doublet having 2p_1/2_ and 2p_3/2_ are 465 and 459.3 eV, respectively, at 0 s, corresponding to Ti-O bond [31]. However, the peaks of 2p_1/2_ and 2p_3/2_ are moved to the left at 2 s due to Ti-N-O bond from the interlayer between TiO_2_ and ZrN [32]. Intermixing could occur between two layers during the deposition of ALD TiO_2_ at 200 °C. Figure 1b shows Zr 3d spectra of TiO_2_/ZrN/Si layers with different etching time. The binding energy peaks for Zr 3d doublet with 3d_3/2_ and 3d_5/2_ are shifted to the left from 2 to 66 s. These peaks are close to the binding energies for the ZrO_2_ at 2 s corresponding to Zr-N-O bond [33,34]. However, the binding energy peaks for Zr 3d_3/2_ and Zr 3d_5/2_ are centered at 182.3 and 179.8 eV, respectively, at 66 s, corresponding to the Zr-N bond [35], which indicates pure ZrN bulk properties for a 30 nm thick ZrN film. Figure 1c shows Si 2p spectra for different sputter etching time. The Si 2p is centered at 99.3 eV, suggesting that the silicon substrate is clearly detected at 136 s [36].

**Figure 2:**
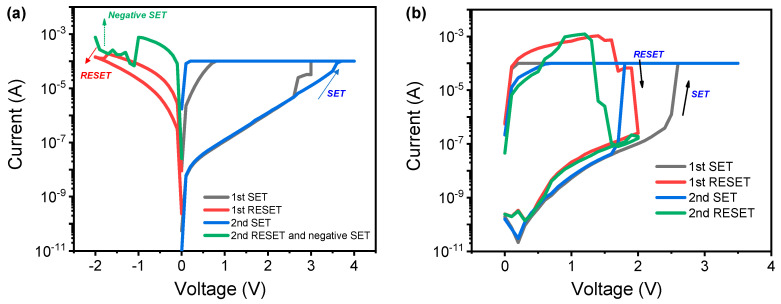
(**a**) Current–voltage (I–V) curves including SET, RESET, and negative-SET of Ag/ZrN/Si device for bipolar resistive switching; (**b**) I–V curves of Ag/ZrN/Si device for unipolar resistive switching.

Figure 2a,b shows the current–voltage (I–V) characteristics of Ag/ZrN/Si device for bipolar and unipolar resistive switching, respectively. SET process enables the transition from high-resistance state (HRS) to low-resistance state (LRS), while the RESET process induces the transition from LRS to HRS. The LRS current is randomly determined during the SET process, implying that Ag migration into ZrN dielectric is uncontrolled even under the same compliance current of 100 μA. The successful RESET process is observed for low LRS (represented by the red color in Figure 2a) for the first RESET process. Negative-SET occurs after the abrupt RESET process at LRS with high current (green color in Figure 2a), which limits the number of reversible cycling between LRS and HRS. Negative-SET makes it difficult to set an appropriate RESET stop voltage by making the margin of the RESET voltage smaller [37]. This phenomenon is coupled with a process in which excessive Ag filaments are formed during the SET process, and the switching fails occur because excessive filaments are formed by parasitic filaments after the RESET process. This process is discussed later, using the filament model. The unipolar switching is also observed as in Figure 2b, where SET and RESET process occurs in the same polarity. The SET process is similar to the Ag filament formation in the bipolar switching by the electric field. However, this RESET process could be quite different from the RESET process in bipolar switching. The level of RESET current is above 1 mA, implying that Joule heating is the dominant switching mechanism. The strong Ag conducting filament could be ruptured by Joule heating when the current flows in the Ag conducting filament. The abrupt RESET switching is generally observed for the rupture process of conducting filament as a result of Joule heating [10].

**Figure 3:**
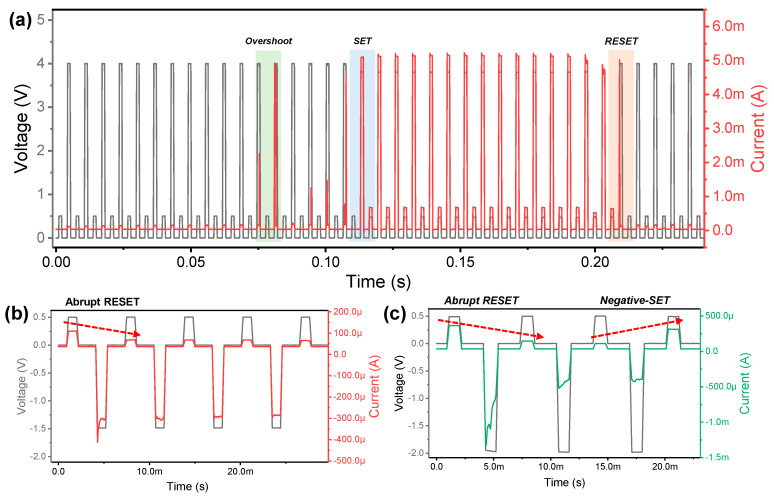
Resistive switching dynamics by pulses, including program/erase and read for Ag/ZrN/Si device. (**a**) Abrupt reset process after abrupt set process by positive voltage pulses. (**b**) Abrupt reset process by negative voltage pulses. (**c**) Negative-SET behavior after gradual reset process.

Next, the transient characteristics of Ag/ZrN/Si device is investigated by voltage pulse for the SET and RESET processes. The current response is monitored when a continuous SET pulse of 4 V and a READ pulse of 0.5 V are applied alternately. Prior to the occurrence of abrupt SET process, a series of overshoot current peaks are observed. In the subsequent multiple pulses, LRS is maintained, while the current suddenly drops for the RESET process. These transient characteristics represent unipolar switching in which SET and RESET is completed in the same polarity. Figure 3b shows the abrupt RESET process when the pulse amplitude of −1.4 V is applied to the device. The lower RESET current is monitored compared to that of the unipolar RESET process. The pulse with larger voltage (−2 V) causes permanent breakdown of the device through negative-SET behavior, which is not desirable for synaptic device in neuromorphic system, as in Figure 3c.

**Figure 4:**
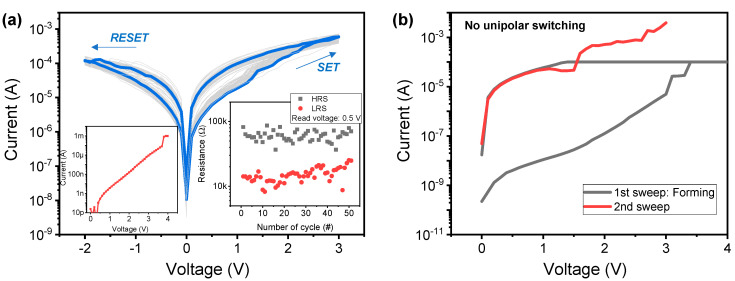
I–V characteristics of Ag/TiO_2_/ZrN/Si device. (**a**) Bipolar resistive switching with gradual SET and RESET processes, forming a curve in the left inset, and endurance cycle in right inset. (**b**) Failure in the working of unipolar switching.

Next, we investigate the basic resistive switching characteristics of TiO_2_ inserted bilayer device (Ag/TiO_2_/ZrN/Si device) for comparison with the single-layer device (Ag/ZrN/Si device). The gradual SET and RESET processes with sub-milliampere level are completed after the electroforming process (Figure 4a). Fifty consecutive DC endurances were achieved in right inset of Figure 4a. It is noteworthy that the variation of switching parameters, including HRS and LRS resistance values in Ag/TiO_2_/ZrN/Si device, is improved compared to the Ag/ZrN/Si device. However, the HRS/LRS ratio is reduced, which is drawback for neuromorphic application.

**Figure 5:**
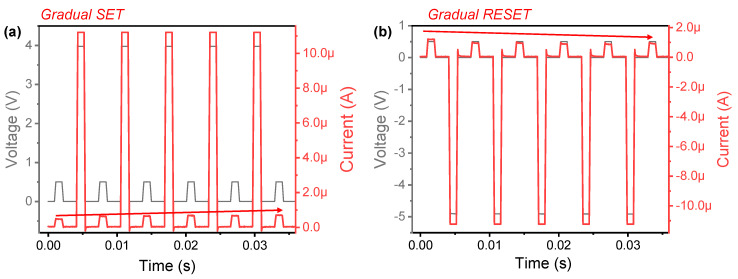
Pulse transients of Ag/TiO_2_/ZrN/Si device for (**a**) gradual SET and (**b**) gradual RESET.

Conversely, unipolar resistive switching does not work in Ag/TiO_2_/ZrN/Si device (Figure 4b). This could be attributed to the very low current for RESET by Joule heating. The gradual SET and RESET switching are also observed through pulse transient, as in Figure 5a,b, respectively. Pulse amplitudes of 4 and −5 V are used for the SET and RESET process, respectively. Notably, the operating currents are significantly reduced despite increased pulse voltage amplitudes for SET and RESET processes in Ag/TiO_2_/ZrN/Si device. In the literature, for the phenomena in which the gradual characteristics are improved according to the additional layer insertion [38,39], the operation polarity change [40] and dielectric thickness control [41] of the device were reported.

**Figure 6:**
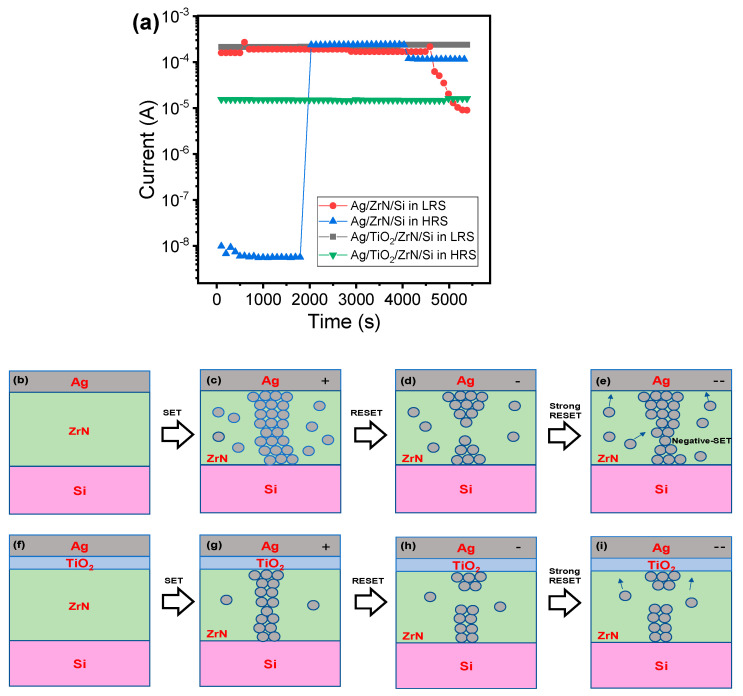
(**a**) Retention test for 5000 s for Ag/ZrN/Si and Ag/TiO_2_/ZrN/Si devices in the low-resistance state (LRS) and high-resistance state (HRS); Ag filament overgrowth and negative-SET in Ag/ZrN/Si device. (**b**) Initial state; (**c**) filament overgrowth in LRS after SET process; (**d**) HRS after the RESET process; (**e**) Additional filament growth through larger RESET voltage; filament evolution for Ag/TiO_2_/ZrN/Si device in the LRS and HRS; (**f**) initial state; (**g**) LRS with smaller size of filament after SET process; (**h**) HRS after RESET process; (**i**) filament is maintained with higher voltage without negative-SET.

Next, the retention test was conducted in order to compare the stability and reliability of Ag/ZrN/Si and Ag/TiO_2_/ZrN/Si device, as in Figure 6a. The LRS and HRS current at 0.2 V of Ag/TiO_2_/ZrN/Si devices is maintained without any significant change for 5000 s. On the other hand, LRS of Ag/ZrN/Si device exhibits very unstable retention behaviors. HRS suddenly changes to LRS at once, suggesting that the Ag clusters aggregate over time, to form a conducting filament. LRS turns to HRS, suggesting that the formed Ag filament is decomposed. The possible bipolar resistive switching mechanisms for Ag/ZrN/Si and Ag/TiO_2_/ZrN/Si devices can be explained by using the filament model. Figure 6b–e shows the Ag filament evolution of Ag/ZrN/Si for SET, RESET, and strong RESET. The initial state (Figure 6b) turns into LRS (Figure 6c) via the SET process. Ag ions move to the cathode (Si substrate) by anodic dissolution, when a positive bias is applied to the Ag top electrode [12]. The Ag conducting filament is formed via the electrochemical metallization effect, once Ag ions reach the Si substrate through the electric field (Figure 6c). RESET process occurs through the dissolution of Ag filament when a negative bias is applied to the Ag top electrode (Figure 6d). Notably, the larger voltage can result in negative-SET behavior in which the Ag clusters can merge, as a result of the large electric field (Figure 6e). The uncontrolled overgrowth of Ag filament during the SET process could result in numerous parasitic filament or clusters. The conducting filament evolution of the Ag/TiO_2_/ZrN/Si device is shown in Figure 6f–i for initial state, LRS by SET, HRS by RESET, and HRS by strong RESET. The smaller size of Ag filament could be formed, considering the lower LRS current in the bilayer device, compared to the single-layer device. Thus, negative-SET behavior is not observed in the case of the strong RESET process, which provides the large RESET voltage margin. The small size of the Ag filament may be a result of the TiO_2_ buffer layer. Similar results of TiO_2_ buffer layer in an Ag/SiO_2_:Ag/TiO_2_/Si device was reported [42]. TiN layer could also slow down Ag diffusion into the dielectric and enhance the threshold switching properties [43].

**Figure 7:**
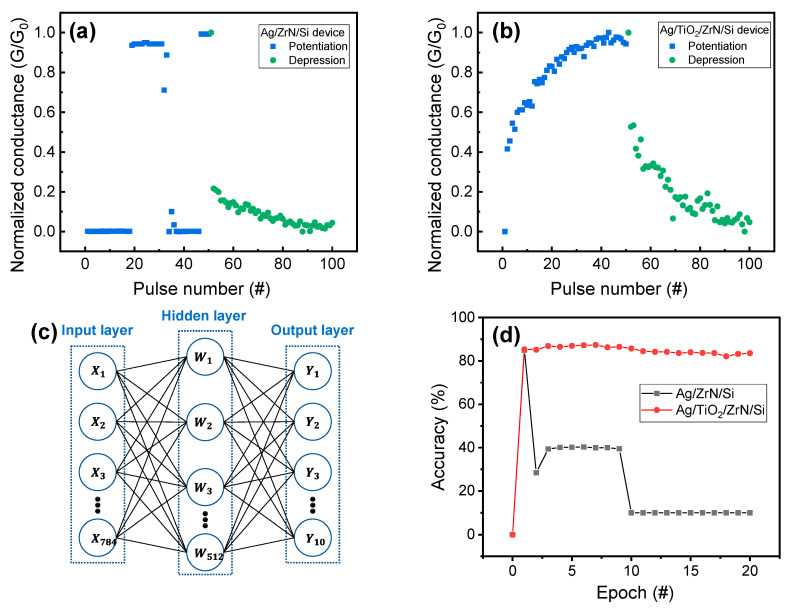
Potentiation and depression of (**a**) Ag/ZrN/Si device and (**b**) Ag/TiO_2_/ZrN/Si device; (**c**) neural network for the pattern recognition accuracy; (**d**) accuracy of Fashion Modified National Institute of Standards and Technology (Fashion MNIST) when applying normalized conductance values, such as synapse array, in a neural network for Ag/ZrN/Si and Ag/TiO_2_/ZrN/Si devices.

Finally, the synaptic performances for Ag/ZrN/Si and Ag/TiO_2_/ZrN/Si devices are compared. The conductance of resistive switching memory can be used for synaptic weight in cross-point array in neuromorphic hardware. Therefore, the multiple conductance values of synaptic device are essential to ensure good neuromorphic performance. The normalized conductance (conductance/peak conductance, G/G_0_) is obtained from measured conductance, at a read voltage of 0.5 V for potentiation and depression of the Ag/ZrN/Si device (Figure 7a). Pulse amplitudes/widths of 4 V/1 ms and −1.5 V/1 ms are used for potentiation and depression, respectively. The transient characteristics for potentiation and depression of Ag/TiO_2_/ZrN/Si device are shown in Supplementary Materials Figure S1.

In potentiation, the uncontrollable conductance changes, such as rapidly increasing and decreasing the conductance, are observed. In the case of depression, the conductance decreases rapidly at the initial point and then gradually decreases. On the other hand, a trend of consistent increase and decrease is observed for the potentiation and depression of Ag/TiO_2_/ZrN/Si devices (Figure 7b). The gradual conductance update is well consistent with the I–V curves presented in Figure 4a. The pulse amplitude and width of 4 V/1 ms and −5 V/1 ms are utilized for potentiation and depression, respectively. In order to evaluate the feasibility of Ag/ZrN/Si and Ag/TiO_2_/ZrN/Si devices for neuromorphic system, a simple neural network is used for pattern recognition (Figure 7c). The neural network consists of input neuron, hidden layer (neuron), and output neuron. The artificial synapse array (resistive switching memory array) are connected between neurons (layers) and the conductance value of resistive switching memory can be represented by the weight of the synapse. For pattern-recognition simulation of the Fashion Modified National Institute of Standards and Technology (MINIST) dataset, 784 × 512 ×10 neurons are used, and the synapses between neurons are densely connected. The Fashion MINIST database consists of 70,000 grayscale images with 10 categories, including coat, bag, and trouser for Fashion images. Then, 28 × 28 grayscale and single channel images are used for input neurons. Input data are normalized to obtain values from 0 to 1 and flattened to a one-dimensional array (784 × 1) for input neurons. We must assume that all neurons are fully connected via resistive switching memory cells as synapses and the normalized conductance values of Ag/ZrN/Si and Ag/TiO_2_/ZrN/Si devices are used as the quantized weights and can be updated by training. The output values are calculated by using vector-matrix multiplication simultaneously, to obtain the pattern-recognition accuracy. Figure 7d shows the accuracy of the pattern recognition as a function of epoch for Ag/ZrN/Si and Ag/TiO_2_/ZrN/Si devices. The bilayer device shows better pattern-recognition accuracy (~87.36%) compared to the Ag/ZrN/Si device. This result demonstrates that Ag/TiO_2_/ZrN/Si device with gradual conductance update is suitable for synaptic device in neuromorphic computing.

## 4. Conclusions

In summary, the current study presents the improved stability and reliability of Ag/TiO_2_/ZrN/Si device, as compared to Ag/ZrN/Si device. First, deposition of ZrN/Si layers is well confirmed through the XPS analysis. The unstable resistive switching, such as negative-SET behavior, is observed in the DC sweep mode and pulse transient for the Ag/ZrN/Si device, due to the overgrowth of Ag conducting filament as a result of current overshoot. On the other hand, gradual SET and RESET switching with enhanced repeatability, variability, and retention are obtained in Ag/TiO_2_/ZrN/Si device by suppressing the migration of excessive Ag. Moreover, in this way, suitable potentiation and depression characteristics for the Ag/TiO_2_/ZrN/Si device are achieved in comparison with the Ag/ZrN/Si device for synaptic device. Finally, a higher pattern-recognition accuracy for the Ag/TiO_2_/ZrN/Si device is achieved by using neural network (784 × 512 ×10) for the classification of Fashion MNIST.

## Supplementary Materials

The following are available online at https://www.mdpi.com/2072-666X/11/10/905/s1. Figure S1: Transient characteristics for (a) potentiation and (b) depression of Ag/TiO_2_/ZrN/Si device.
