# Supplementary material for: Improved Stability and Controllability in ZrN-Based Resistive Memory Device by Inserting TiO2 Layer
Source: Micromachines (Basel). 2020 Sep 29;11(10):905. doi: 10.3390/mi11100905 (PMC7600328; doi:10.3390/mi11100905)
Supplement: Supplementary file 1 [file micromachines-11-00905-s001.pdf]

Supplementary Materials: Improved Stability and Controllability in ZrN-Based Resistive Memory Device by Inserting TiO_2 Layer

Junhyeok Choi and Sungjun Kim

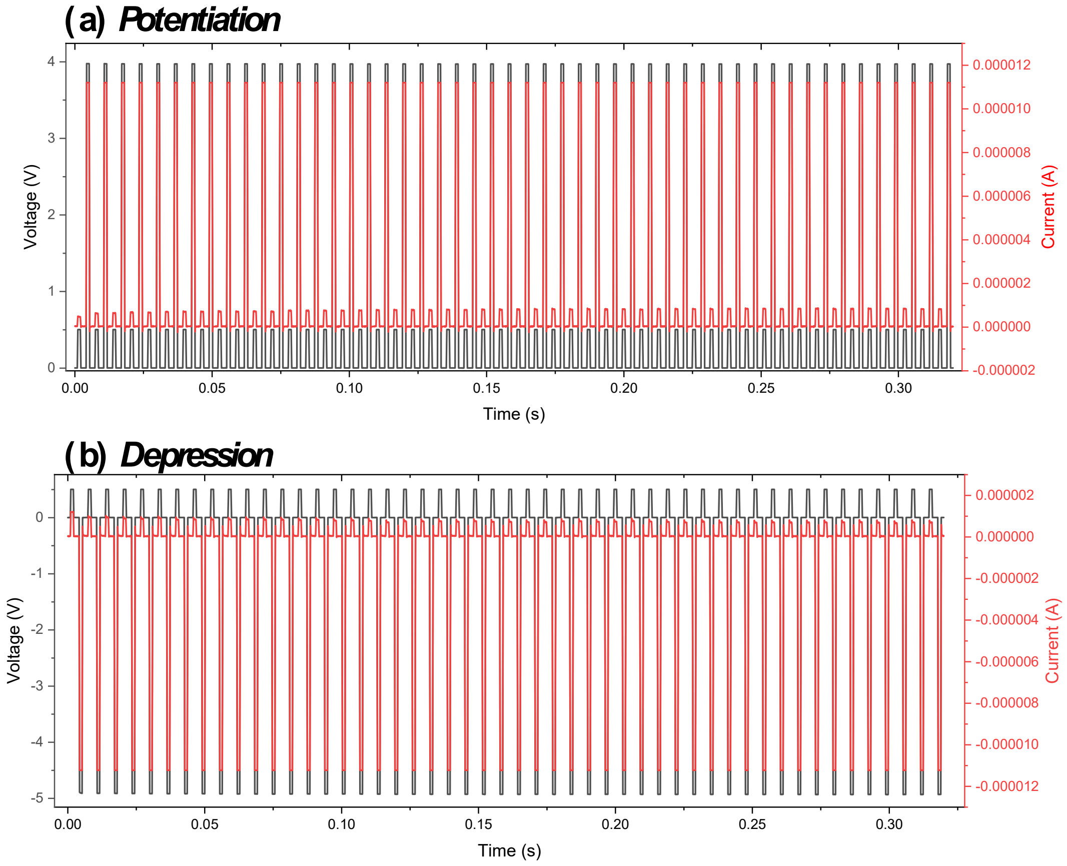

Figure S1. Transient characteristics for (**a**) potentiation and (**b**) depression of Ag/TiO2/ZrN/Si device.
